# A Case of the Heroin Habit
1Read at a Meeting of the Bath and Bristol Branch of the British Medical Association, March 25th, 1912.


**Published:** 1912-06

**Authors:** J. Odery Symes

**Affiliations:** Physician to the Bristol General Hospital


### A CASE OF THE HEROIN HABIT.[1]

BY

J. ODERY SYMES, M.D.,

*Physician to the Bristol General Hospital.*

---

HEROIN, or diacetyl morphia, is chiefly used in this country as a sedative in cases of troublesome cough, and is generally given as the hydrochloride in a mixture. I am informed, however, by Messrs. Burroughs and Wellcome, that it is largely used at the present time hypodermically, chiefly, I imagine, for the relief of pain or the control of asthma. It is desirable, therefore, that medical men should know that there is a grave risk of a patient using this drug contracting a heroin habit similar to the morphia habit. Several French writers[1] have drawn attention to this point, and from their experience with cases of heroin mania they are convinced that the effects of this drug are more profound than those of morphia, and that the suppression of the habit is more difficult, more dangerous, and more painful.

Duhem, who reports upon seventeen cases of heroin mania, says that in the process of cure the chief danger is that of

[1] Duhem, *Progrès méd.*, 1907, xxiii. 113; *Tribune méd.*, July 1st, 1905.

[1] Read at a Meeting of the Bath and Bristol Branch of the British Medical Association, March 25th, 1912.

respiratory failure, which comes on without any preliminary failure of the heart. Respiratory failure is particularly apt to occur in cases in which the drug is withdrawn too rapidly. Amongst other symptoms during the period of cure he notes hallucinations and delirium, periods of emotional excitement, sleeplessness, headache, generalised pain, and the very slow return to normal health and weight. The dose of heroin in the cases reported varied from 1 to 5 grains a day.

A case has recently come under my care in which the patient was supplied by a medical man with a hypodermic syringe and a supply of heroin hydrochloride tabloids to be used for the relief of pain, and was assured there was no danger of contracting a habit from its continued use. The patient, a lady of 39 years of age, whilst staying at a well-known spa, was seized with neuritis in the right arm. As other remedies failed to give relief, the doctor gave a hypodermic injection of $\frac{1}{12}$ grain of heroin hydrochloride, and directed that the nurse should repeat the dose whenever the pain recurred. The first effect of the injection was to relieve the pain, but half an hour later vomiting commenced and continued during the night. This was late in 1906. As the pain continued, the injections were repeated, first by the doctor, then by the nurse, and later by a relative, and the dose was gradually increased to $\frac{1}{4}$ grain three or four times a day. During the next two years the patient made ineffectual attempts to break off the habit. In 1907 she learned to use the hypodermic syringe herself. From this time the habit gradually became more confirmed, and the dose increased, until at the time of her breakdown, in October, 1911, she was taking between 4 and 5 grains in the twenty-four hours. The daily allowance never exceeded 5 grains.

With regard to the effects of the drug, I cannot do better than describe some of these in the patient's own words, for she is an observant woman of education and refinement. Even after the first dose there was " a feeling of excessive fidgetiness, which seemed to creep right up from the very lowest part of the body (the womb), making me so terribly restless that I could not keep my legs still for a moment. This particular feeling increased maddeningly until I had the injection again, when I felt immediately relieved and blissfully comfortable for about $2\frac{1}{2}$ to 3 hours." It was the recurrence of these feelings which led to the repetition of the drug throughout. Gradually the patient's health began to fail ; she became thinner, less able to take physical exertion, depressed and seedy. The appetite was very poor, and she noticed a peculiar salt taste in the mouth when the drug was withheld ; occasionally she had attacks of vomiting, and constipation was most troublesome. The catamenia became at first irregular and scanty, and finally ceased. She had never been a good sleeper, but since taking heroin this had become much worse, and she had recourse to drugs (chiefly veronal) to induce sleep. If the interval between the injections of heroin was too long she "felt wretchedly fidgety and uncomfortable, and people and things seemed to go farther from me as I looked at them, and at times I felt almost light-headed." If the dose was increased too rapidly it gave rise to palpitation and excitement. Amongst other symptoms noticed were increased sensibility to sounds and loss of recuperative power after small injuries, such as cuts ; another most troublesome symptom was difficulty in starting micturition. There were never any symptoms of respiratory failure, oppression of breathing, etc. The patient says that whilst taking the drug (although previously extremely subject to colds) she never sneezed or coughed, nor did she ever catch a cold during the whole five years.

The relief given by the injections was instantaneous, but these had to be repeated at shorter and shorter intervals. The patient describes herself as having felt "bucked up," both physically and mentally, keener sensed, calmer, happier in mind, and more ready and able to undertake the exertion entailed by golf, bicycling, roller skating, or walking immediately after taking the drug. There was no marked mental or moral failure. Finally, in October, 1911, when taking about 5 grains of heroin a day, she was seized with a violent attack of vomiting and prostration with alternately shivering and perspiring. She was unable to move from her bed, and the habit being discovered, an attempt was made to break it off by gradually tapering the dose. Six weeks from the commencement of treatment the dose had been reduced from 5 grains to $\frac{4}{50}$ths of a grain a day. The chief difficulties had been sleeplessness, restlessness, emotional outbreaks, tachycardia, loss of appetite and prostration. The patient was at the time walking out of doors, and the mental condition was normal. Any attempt to reduce the heroin beyond $\frac{4}{50}$ths of a grain, although unknown to the patient, gave rise to great discomfort and increased emotional excitement. I therefore substituted $\frac{1}{8}$th grain of morphia twice a day, and decreased this amount daily. With the substitution of morphia the restlessness disappeared, and by the end of the eighth week hypodermic injections of sterile water only were employed. During the last week of the cure she suffered from a severe cold, the first for five years.

At the present time, six months from the commencement of the cure, the patient is in splendid health, and professes to have no craving for the drug. Sleeplessness, which has been a troublesome symptom throughout the cure, is relieved by the use of adalin, and this drug is still occasionally used.

